# Frozen Elephant Trunk Technique in Acute Type A Aortic Dissection: Is It for All?

**DOI:** 10.3390/medicina57090894

**Published:** 2021-08-28

**Authors:** Pierpaolo Chivasso, Generoso Mastrogiovanni, Mario Miele, Vito Domenico Bruno, Antonio Rosciano, Antonio Pio Montella, Donato Triggiani, Mario Colombino, Francesco Cafarelli, Rocco Leone, Paolo Masiello, Severino Iesu

**Affiliations:** 1 Department of Emergency Cardiac Surgery, University Hospital San Giovanni di Dio e Ruggi d’Aragona, 84126 Salerno, Italy; genemas60@yahoo.it (G.M.); mariomiele@live.it (M.M.); ant240794@gmail.com (A.R.); ap.montella@gmail.com (A.P.M.); donato.triggiani@sangiovannieruggi.it (D.T.); mario.colombino@sangiovannieruggi.it (M.C.); francesco.cafarelli@sangiovannieruggi.it (F.C.); rocco.leone@sangiovannieruggi.it (R.L.); paolo.masiello@sangiovannieruggi.it (P.M.); severino.iesu@alice.it (S.I.); 2 Translational Health Science Department, Bristol Medical School, University of Bristol, Bristol BS8 1TH, UK; vitodomenicobruno@gmail.com

**Keywords:** type A aortic dissection, aortic arch surgery, FET, frozen elephant trunk, hybrid arch surgery

## Abstract

Acute type A aortic dissection (ATAAD) is an indisputable emergency with very poor outcomes without surgical treatment. Although the aortic arch is often involved in the aortic dissection, its optimal management during surgical therapy remains uncertain. A conservative tear-oriented approach has traditionally been adopted, limiting the procedure to the ascending aorta (or hemiarch) replacement. However, dilation of the residual dissected aorta and subsequent rupture may occur, requiring further intervention in the future. In the last two decades, the frozen elephant trunk (FET) technique has become a valid and attractive option to treat aortic disease when the arch and the thoracic aorta are involved, both in elective and in emergency settings. Here, we report a review of the contemporary literature regarding the short- and long-term outcomes of the FET technique in ATAAD repair.

## 1. Introduction

Acute type A aortic dissection (ATAAD) is one of the most complicated and life-threatening conditions of the cardiovascular system. Aortic rupture and consequent cardiac tamponade are the most common cause of death. Emergency surgery, in most of the cases, is the only therapeutic option [1,2]. Although advances in surgery techniques have improved the outcomes for patients with acute dissection, the largest registries indicate a persistently high operative mortality of 15–20% [3,4,5]. Consequently, a conservative approach aiming at performing the simplest and straightest operation with the minimum impact on the patient, such as ascending aorta or hemiarch replacement, has traditionally been adopted [6]. However, dilation of the residual dissected aorta and subsequent risk of rupture requiring further intervention in the future may occur. In the last two decades, the frozen elephant trunk (FET) technique has become a valid and attractive option to treat aortic disease when the arch and the thoracic aorta are involved, both in elective and in emergency settings [7,8,9]. As reported by Aftab andcolleagues [10], the FET technique converts the conventional elephant trunk procedure, which is inherently a two-stage operation, into a one-stage repair by completely replacing the aortic arch with a surgical prosthesis which comprises a distal stented portion deployed into the distal tract of the aortic arch and the proximal descending thoracic aorta (DTA). Particularly, in acute aortic dissection, the use of FET can help to expand and stabilize the true tear and cover eventual supplementary ones in the stented portion of the aortic arch or proximal DTA [11]. Despite these potential advantages, an extensive use of this technique has been limited by the higher surgical trauma it involves for the patient and the alleged technical difficulties for the surgical team performing the operation. For this reason, only a limited number of institutions have adopted this procedure to treat aortic dissection, based on the assumption that it may be too complicated in an emergency setting.

At present, there is not enough evidence accounting for the clinical significance of replacing the aortic arch as an additional procedure during emergency proximal aortic repair for ATAAD, thus making comparison of this technique’s outcomes with those of secondary total arch replacement problematic [12].

## 2. Preoperative Considerations and Surgical Procedure

As stated by Peterss and colleagues [13], it is not easy to apply the principle ‘keep it simple and smart’ to the difficulties and complexities of aortic arch surgery. Total arch replacement with the FET technique in patients with ATAAD is a challenging operation requiring substantial experience in surgical treatment for acute dissection as well as the best possible strategies for organ protection. In this context, a perfect and standardized planning of the surgical procedure is essential. The first step of a successful therapy is an exact evaluation of the aortic anatomy from a preoperative angio-computed tomography (CT) scan, which can carefully assess the exact location of the entry and re-entry tears, the origin of visceral vessels distributed between the true and the false lumen, the extent of the dissecting process, and the aortic diameters [6].

There are several prostheses which can be used for FET interventions [14]. The most popular and frequently used are the Thoraflex hybrid prosthesis (Vascutek, Inchinnan, Scotland) and the E-vita Open Plus prosthesis (JOTEC GmbH, Hechingen, Germany) [14]. Both are considered hybrid, as they have a proximal vascular graft (quadrifurcated in the Thoraflex and tubular in the E-vita Open Plus) attached to a distal stent graft. Both these prostheses are available in different sizes and are commonly used in acute dissection and other diseases involving the arch and/or the DTA.

**Figure 1:**
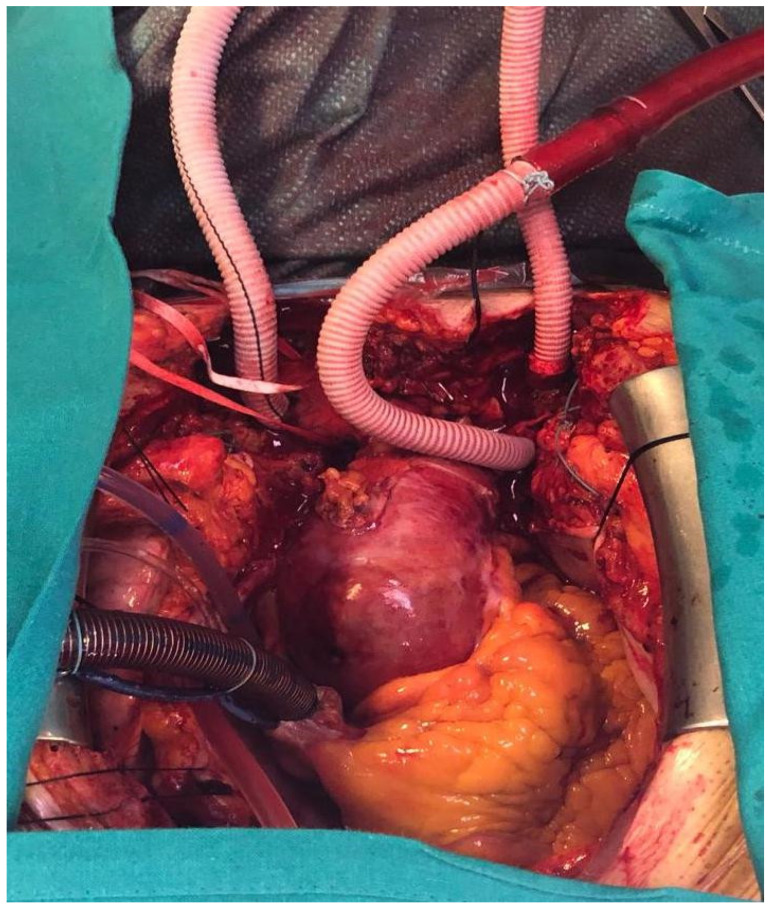
Selective cannulation of intrathoracic right subclavian (RSA), left common carotid (LCA), and left subclavian arteries (LSA) with interposition of an end-to-side dacron vascular graft.

In our institution, we largely use the Thoraflex hybrid. The main surgical steps include: intra-thoracic right subclavian artery (RSA) cannulation through interposition of a 10 mm end-to-side dacron vascular graft; debranching of the left common carotid (LCCA) and left subclavian (LSA) arteries and their cannulation through interposition of vascular grafts (debranching first approach with complete “trivascular” brain perfusion by the three supra-aortic vessels, as shown in Figure 1); total resection of the aortic arch; preparation of the distal aortic stump and obliteration of the false lumen using three semicontinuous polypropylene 3/0 stitches with Teflon felts both inside and outside to re-compact the dissected aortic wall; stent graft deployment of the Thoraflex hybrid system; fixation and suturing of the graft to the distal stump (usually in zone 2) and arch replacement with the proximal vascular segment of the hybrid prosthesis. The arch vessels are separately reimplanted to branch grafts of the prosthesis.

The choice of the stented graft size is determined by the aortic diameter of the landing, zone which is evaluated by a preoperative CT angiogram of the aorta. In case of acute aortic dissection, no oversizing is performed to prevent the formation of new intimal tears distal to the stent graft, which can potentially promote retrograde perfusion of the false lumen around the stented portion of the prosthesis, thus impeding aortic remodeling of the DTA.

## 3. Cannulation Strategy

Cardiopulmonary bypass (CPB) and its initiation can be achieved with different approaches and by cannulating different vessels, each of them providing definite risks and benefits which should be taken in account when deciding which strategy to use [13].

The use of the axillary or the subclavian artery as the entry site for the arterial line of the CPB, either by direct cannulation or by interposition of an end-to-side graft, has gained increasing popularity, having proven to be safe also in ATAAD with structural involvement of the innominate artery. This approach offers a safe antegrade flow throughout the entire procedure and helps to deliver cerebral perfusion through the primary cannulation site in a unilateral approach during selective cerebral perfusion. Moreover, using axillary or subclavian artery as the site of cannulation, it is possible to initiate CPB in a no-touch manner with regard to the aorta, limiting its manipulation and the consequent risk of dislodgement of embolic debris or rupture.

A more traditional way to initiate CPB is by cannulating the femoral artery via an open Seldinger-guided technique. This approach offers early reperfusion of the lower body via retrograde perfusion flow once the distal anastomosis is complete [15]. However, in contemporary practice, this perfusion strategy is not largely employed anymore, as there is large evidence that retrograde flow can potentially lead to emboli from abdominal and descending aortic debris, retrograde dissection, and also malperfusion syndrome [16,17].

Direct aortic cannulation has also been described as an alternative approach to preserve the antegrade flow throughout the aorta, thus sparing time by avoiding additional incisions and dissection of different vessels such as the axillary or subclavian artery [16]. However, the direct manipulation of a dissected ascending aorta can raise the risk of rupture and dislodgement of calcium or debris. Moreover, because of the difficulty of recognizing the true lumen, it is possible to cannulate the false lumen by mistake, thus causing systemic malperfusion, impacting the outcome.

As previously reported by our group [18], in our institution, the right intrathoracic subclavian artery is routinely employed as the site for central cannulation. Its exposure is facilitated by extending the usual sternotomy incision in a small right supra-clavicular cervicotomy. In all cases, an end-to-side dacron vascular graft (8 or 10 mm, depending on the native vessel size) is interposed to avoid the direct cannulation of the artery.

## 4. Cerebral, Spinal Cord, Viscera, and Heart Protection

**Figure 2:**
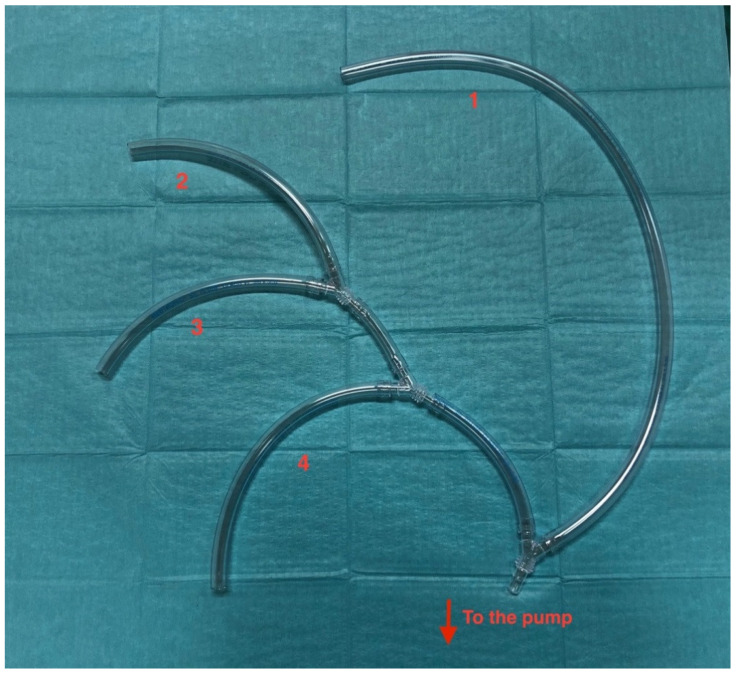
Home-made perfusion system driven by a single pump: 1, right intrathoracic subclavian artery perfusion (systemic perfusion); 2, left subclavian artery perfusion; 3, left carotid artery perfusion; 4, side-branch systemic perfusion [18].

Organ protection involves advanced strategies which allow increasing tolerance to ischemia of the brain, spinal cord, myocardium, and visceral organs. There is a large body of evidence supporting the principle that selective antegrade cerebral perfusion (SACP) according to the Kazui’s technique for brain protection is a valid option to achieve an effective cerebral protection during the phase of circulatory arrest [19,20]. This strategy, relying on the integrity of the circle of Willis, is commonly adopted together with moderate hypothermia, lowering patient’s nasopharyngeal temperature to 26–28 °C. However, it is well known that anatomic variations may occur, leaving a considerable number of patients still at risk for neurologic injuries despite the SACP strategy [21]. Considering that in an emergency setting it is not routinely possible to investigate whether the circle of Willis is complete, to reduce the risk of incomplete or sub-optimal brain protection, we use to establish a circulatory arrest and, through the cannulated right subclavian (or axillary), LCC, and LS arteries, complete “trivascular” brain perfusion is initiated at a flow rate of 10 to 15 mL/kg/min and is adjusted to maintain the left femoral artery pressure between 40 and 70 mmHg. The cardiopulmonary bypass (CPB) and SACP are performed using a homemade circuit (Figure 2) which consists of four branches of the same diameter driven by a single pump; these branches are used to perfuse LSA, LCCA, brachiocephalic artery, and the prosthetic branch for systemic perfusion. The perfusion is kept at full flow for CPB, and re-distributes depending on the physiological systemic resistance. When the brachiocephalic artery is clamped, the flow is lowered to 10–15 mL/Kg/min for isolated cerebral perfusion. After the circulatory arrest time, the full flow is restarted via prosthetic side branch and single cerebral vessels. Of course, we are aware that unilateral or bilateral SACP is commonly employed by other centers in Europe with comparable post-operative stroke rates. However, we believe that a “trivascular” perfusion, especially throughout the phase of circulatory arrest, may lead to a lower rate of neurological and spinal injuries, as demonstrated by our initial experience with the FET technique even in emergency cases (paper in press).

The main general principles for spinal cord and visceral protection include the use of hypothermia, perfusion of the three supra-aortic vessels (“trivascular” perfusion) always including LSA, and a strategy that we have recently adopted, i.e., a prompt antegrade reperfusion of the thoraco-abdominal aorta via the arch graft as soon as the distal anastomosis is started, with a blood flow of 1200 mL/min, which reaches the systemic flow once the distal anastomosis has been completed.

As reported by Berger and colleagues, myocardial protection can be achieved in different ways [22]. While cold blood cardioplegia in an antegrade and/or retrograde fashion can be effectively used in all scenarios, in selected patients, providing that cross-clamping is safely feasible, it is possible to achieve myocardial protection by using continuous normothermic selective coronary perfusion at a rate of 100–200 mL/min through the cardioplegia line, as described by the Hannover group [7]. In our institution, Custodiol (HTK–Custodiol; Koehler Chemi, Alsbach-Haenlien, Germany) is generally used for myocardial protection. With a single dose of 20 to 25 mL/kg, Custodiol infusion guarantees a safe myocardial ischemic time of 180 min. This is infused into the aortic root when feasible or directly into both coronary ostia. More rarely, we have administrated it retrogradely through the coronary sinus.

## 5. Methods

A systematic literature search was conducted through PubMed for any study published in the last 13 years on aortic dissection; an additional search was performed in the same period of time, aiming at studies evaluating the surgical outcomes of type A aortic dissection repair with the FET technique. We searched the term [“Aortic Dissection” (all fields)] and a combination of it with the terms: [FET (all fields)], {FET [MeSH term]}, [typeA dissection(all fields)], {typeA dissection [MeSH term]}, [aortic arch(all fields)], {aortic arch [MeSH term]}, [aortic arch surgery (all fields)], {aortic arch surgery [MeSH term]}. To further identify the papers of interest, a cross check with the same searching criteria was conducted on Scopus. Two reviewers (P.C. and G.M.) independently assessed the online databases (last access on the 9 July 2021), screening titles and abstracts. The full-text articles were then obtained for all potentially eligible articles that clearly met the inclusion criteria and were reviewed separately if either reviewer considered the manuscript as being eligible. Any disagreements were resolved by consensus. Case reports, editorials, reviews, and meta-analyses were excluded. Non-clinical or post-mortem reports were also excluded. The selection inclusion criteria were: (I) articles addressing acute type A aortic dissection treated with the FET technique; (II) articles including the use of hybrid prostheses; (III) articles reporting short-term mortality and/or post-operative stroke rate and/or mid- long-term follow-up. All publications were limited to human subjects and written in English. All data were independently extracted from the studies by two investigators (P.C. and V.D.B.). The final results were reviewed by the senior reviewer (S.I.). Our main outcomes of interest were short-term post-operative mortality, post-operative stroke, and spinal cord injury. Data on those main outcomes were retrieved from the original articles. Short-term mortality was defined as in-hospital or 30-day post-operative mortality. In cases where both time points was available, only the in-hospital mortality was used. Stroke was defined as permanent post-operative neurological events as stated by the papers. Further outcomes at follow-up were also considered, such as mortality, false lumen thrombosis as shown by follow-up CT scans, and distal aorta reoperations.

## 6. Results

**Figure 3:**
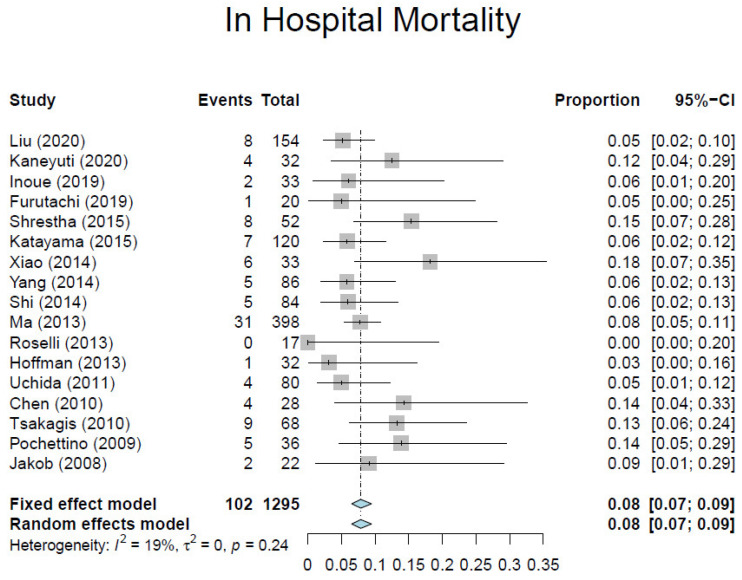
Forest plot depicting pooled estimates of hospital mortality.

**Figure 4:**
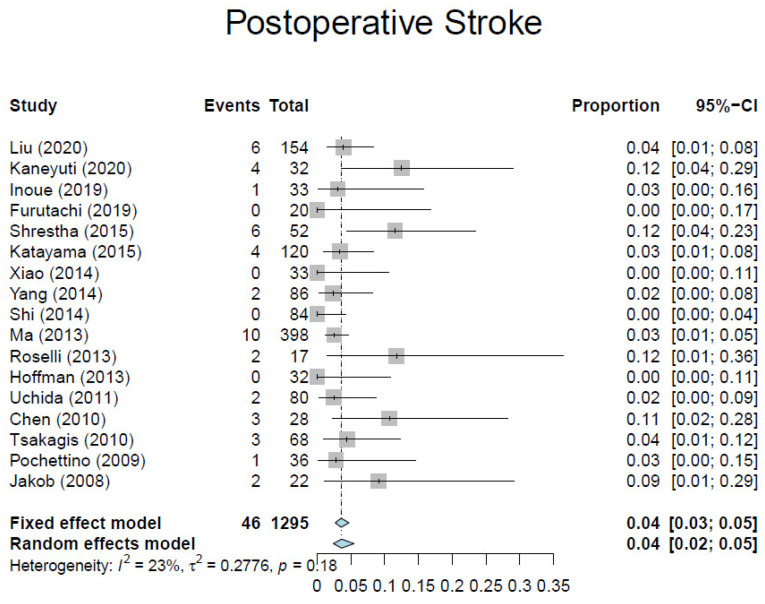
Forest plot depicting pooled estimates of stroke.

**Figure 5:**
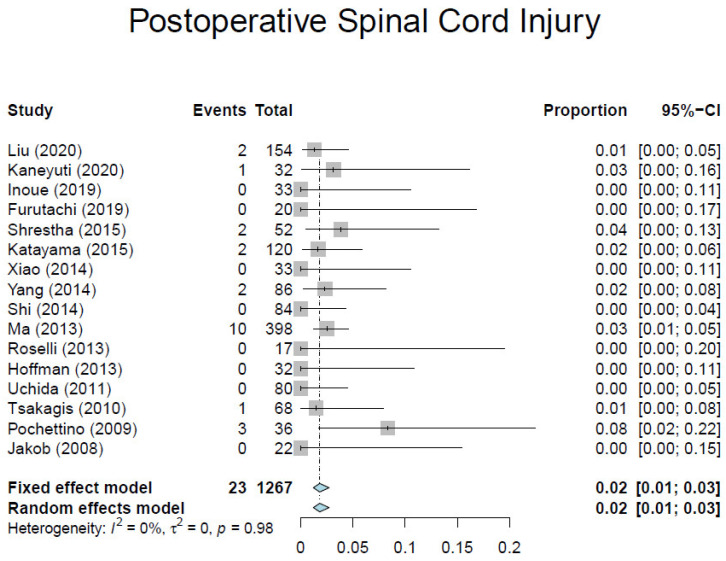
Forest plot depicting pooled estimates of spinal cord injury.

Based on our criteria, we identified 17 observational studies [7,10,23,24,25,26,27,28,29,30,31,32,33,34,35,36,37] involving 1295 patients (Table 1). Overall, the average weighted in-hospital mortality was 7.8%, ranging from 0% to 18.2% (Figure 3). Post-operative stroke occurred in 3.5% of patients (range, 0–13.0%) (Figure 4), and spinal cord injury occurred in 1.7% of patients (range, 0–9%) (Figure 5). These positive trends should be viewed with caution, as these studies were conducted by experienced aortic surgeons working in high-volume centers.

**Figure 6:**
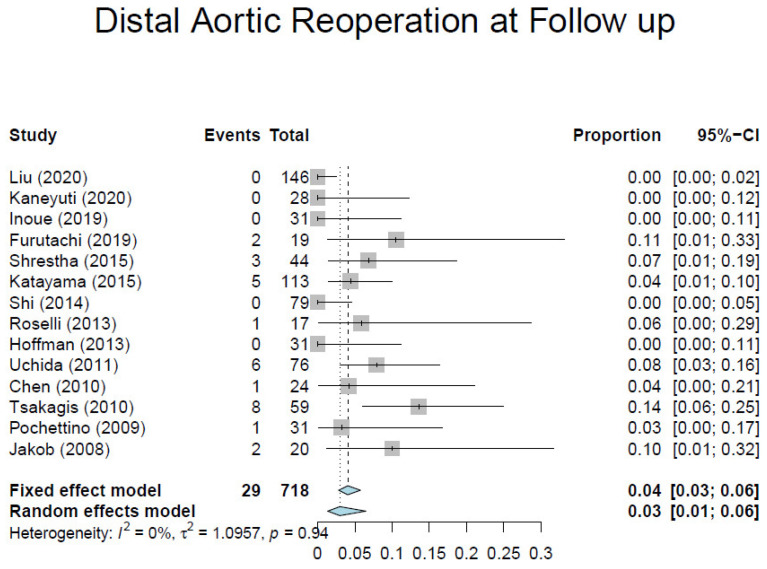
Forest plot depicting pooled estimates of distal aortic re-operation at follow-up.

**Figure 7:**
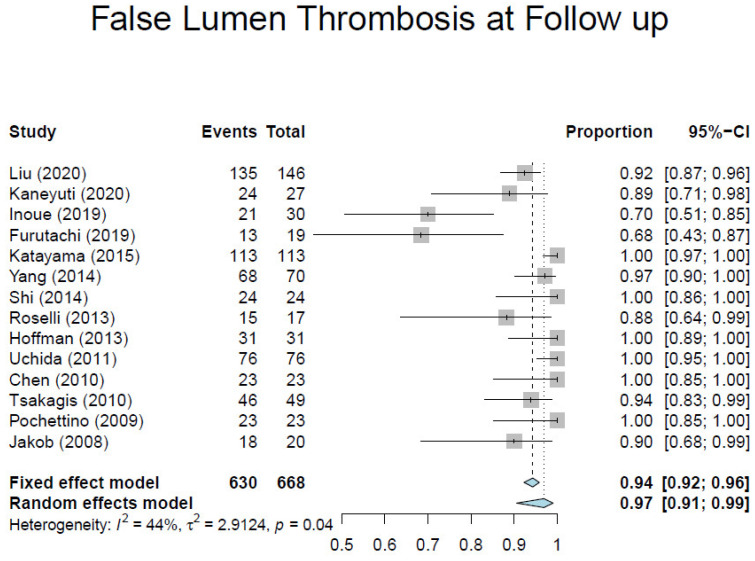
Forest plot depicting pooled estimates of false lumen thrombosis at follow-up.

Table 2 shows the mid- and long-term outcomes. Long-term data from contemporary literature presented significant discrepancies, and only few studies showed a mean follow-up longer than 1 year, thus making the analysis of these results less consistent. One-year survival ranged from 80% to 100%, and 5-year survival from 68% to 96%. The pooled average of 1-year freedom from aortic re-operation was 95.1% (range, 86.5–100%) (Figure 6). Pooled average aortic remodeling, defined as partial or complete thrombosis of the false lumen around the stented portion of the DTA, was 92% (Figure 7). These data compare favorably with those reported by the largest registries for conservative management, which range from 33.3% to 77.8%, thus suggesting that favorable aortic remodeling is likely to happen after FET [6,23,24,25,26,27]. Interestingly, Chinese patients seem to do better than American and European patients undergoing ATAAD repair with the FET technique. These patients may have better outcomes because they are younger and possibly due to natural characteristics which could represent an intrinsic bias.

## 7. Discussion

In the last decade, several studies have been published demonstrating good early outcomes of the FET technique in the treatment of ATAAD when performed by experienced surgeons at high-volume centers compared to large registry results of conventional repair.As reported by Schrestha and colleagues, in a recent position paper by the Vascular Domain of EACTS (European Association of CardioThoracic Surgery), the most frequent post-operative complications were stroke, spinal cord injury (SCI), and acute kidney injury (AKI) requiring dialysis [43]. The main problem with the description of post-operative outcomes is the lack of uniformity in defining and reporting complications. Nevertheless, we focused our review on in-hospital mortality and cerebrovascular and spinal events.

There is a large ongoing debate in the cardiac surgery society concerning the most appropriate management of the aortic arch in ATAAD and, particularly, whether the FET technique should be used routinely to treat these patients, regardless of the primary involvement of the arch itself in the pathologic process. The less recent literature advocated that because of significant additional hazards, total arch replacement should not be routinely performed unless there is a clear indication such as a dilatation of the arch or the presence of an intimal tear clearly detected by a pre-operative CT angiogram [1,2]. However, in the last two decades, more extensive surgical procedures including total arch replacement have been adopted, demonstrating a reduction in the need of subsequent aortic re-interventions and improving long-term outcomes at follow-up [27]. In our opinion, the claimed technical challenge of this technique should not represent a limit to its use in acute settings. The contemporary concept of specialized centers with a high volume of aortic surgery treating both chronic and acute aortic syndrome is now becoming dominant and possibly changing the surgical approach [18]. There is a substantial unanimity on the idea that patients affected by acute aortic syndromes may well gain advantages from treatment at dedicated specialized aortic centers, with significantly improved results and decreased mortality [44,45,46]. We think that the future treatment of ATAAD is moving toward a total arch approach with standardized cerebral protection techniques, delivered by specialized high-volume aortic centers.

Aortic arch aneurysm and the evidence of an intimal tear or retrograde dissection should be deemed as indications for a surgical treatment including total arch replacement. The potential benefits of extended arch surgery in patients with ATAAD are the resection of primary intimal tears beyond the ascending aorta when detected, the exclusion of re-entry tears in the distal aorta by delivering the stented portion of the hybrid prosthesis, the re-expansion of the distal true lumen, and indorse false lumen obliteration [47]. Particularly, in patients presenting with distal aorta malperfusion due to true lumen compression by the false lumen, the FET technique can potentially entirely re-expand the compressed true lumen as well as cover additional entry tears at the level of the proximal DTA, which may well sustain pressurization of the false lumen [48]. In addition, there is evidence that the natural history of the distal false lumen of the aorta following a primary procedure of ascending aorta or hemiarch replacement may lead to degeneration, aneurysm, and/or rupture, thus requiring additional extensive interventions. Data from large registries and recent literature suggest that in 60% to 90% of cases, a negative remodeling of the distal aorta takes place, to the extent of requiring second-stage endovascular or open surgical completion [24,25,26,27]. In acute dissection, the FET technique is expected to diminish DTA dilatation by allowing both coverage of secondary entry tears located in the proximal DTA and obliteration of the false lumen at the proximal DTA, thus decreasing aortic-related deaths and the need for challenging distal aortic reinterventions.

From a technical point of view, we believe that FET hybrid prostheses make aortic arch surgery easier in this context, by avoiding the need of completing a challenging distal anastomosis deeply at the level of the proximal DTA, where there is a huge risk of bleeding or rupture mainly due to the fragile dissected aortic wall. This aspect has been underlined by several surgeons who have systematically adopted this strategy in emergency settings. As reported by Di Bartolomeo and colleagues, taking advantage of the distal stent graft segment of the FET prosthesis, a distal anastomosis can be more easily performed at a more proximal level (generally, in zone 2 of the aortic arch, proximally to the left subclavian artery), while still excluding the distal arch tear [6]. It is also important to mention that the total arch and frozen stented elephant trunk technique avoids the learning curve of endovascular technology by allowing the surgeon to deploy the distal stent graft during circulatory arrest under direct view, thus avoiding the use of fluoroscopy at the time of an emergency operation. Although the descending aorta cannot be inspected by the surgeon operating by way of a median sternotomy, stent graft placement in an antegrade fashion into the downstream aorta can be safely performed [40].

For all these reasons, although stronger evidence of long-term benefits is still needed, we believe that in an early future, all patients presenting with ATAAD should be at least considered for surgical treatment using the FET technique. Surely, the condition of the patient must be taken in account in the process of deciding which approach should be adopted to treat this condition. In fact, even if the anatomy of the dissection is suitable, total arch replacement may be not indicated, such as in elderly patients or in particularly poor clinical conditions with signs and symptoms of systemic malperfusion [22].

## 8. Conclusions

The surgical management of ATAAD still remains a great challenge, also in expert hands. The FET technique seems to be associated with favorable early results. When used by experienced and capable surgical teams, it appears to be associated with acceptable in-hospital mortality and stroke rates, and the risk of spinal cord injury seems to be lower when compared to large registry results of conventional repair. Moreover, by promoting the positive remodeling of the distal aorta, the FET technique is likely to improve patients’ long-term survival and decrease their need for secondary procedures in the future.

## Figures and Tables

**Table 1:** Early outcomes after extensive ATAAD repair with Fthe ET technique.

Study	Year	Country	N. of Patients	Male n.(%)	Age (Mean)	Hospital Mortality n. (%)	Stroke n.(%)	SCI n.(%)
Liu [28]	2020	China	154	93 (60.3)	52.5	8 (5.19)	6 (39)	2 (1.3)
Kaneyuti [29]	2020	Japan	32	25 (78)	59	4 (13)	4 (13)	1 (3)
Inoue [30]	2019	Japan	33	25 (75.7)	67	2 (6.1)	1 (3)	0
Furutachi [31]	2015	Japan	20	15 (75)	58.8	1 (5)	0	0
Shrestha [7]	2015	Germany	52	8 (2.7)	59	8 (15.4)	6 (11.5)	2 (3.8)
Katayama [32]	2015	Japan	120	64 (53.3)	64	7 (5.8)	4 (3.3)	2 (1.7)
Xiao [33]	2014	China	33	24 (72.7)	48	6 (18.2)	0	0
Yang [34]	2014	China	86	69 (80.2)	45	5 (5.8)	2 (2.3)	2 (2.3)
Shi [35]	2014	China	84	57 (67.9)	54	5 (5.9)	0	0
Ma [36]	2013	China	398	328 (82.4)	46	31 (7.8)	10 (2.5)	10 (2.5)
Roselli [10]	2013	USA	17	14 (82)	61.4	0	2 (12)	0
Hoffman [37]	2013	Germany	32	26 (81.2)	58	1 (3.1)	0	0
Uchida [38]	2011	Japan	80	36 (45)	57	4 (5)	2 (2.5)	0
Chen [39]	2010	China	28	22 (78.6)	51	4 (14.3)	3 (10.7)	NA
Tsakagis [40]	2010	Germany	68	52 (76.5)	68	9 (13.2)	3 (4.4)	1 (1.5)
Pochettino [41]	2009	USA	36	NA	59	5 (14)	1 (3)	3 (9)
Jakob [42]	2008	Germany	22	17 (77.3)	57	2 (9.1)	2 (9.1)	0

ATAAD: acute type A aortic dissection; FET: frozen elephant trunk; SCI: spinal cord injury.

**Table 2:** Outcomes at follow-up after extensive ATAAD repair with the FET technique.

Study	Year	Country	Mean Follow Up (Months)	False Lumen Thrombosis n. (%)	Distal Aortic Reoperation n. (%)	Mortality at Follow Up n. (%)
Liu [28]	2020	China	21	135 (92.4)	0	13 (8.9)
Kaneyuti [29]	2020	Japan	19	24 (88.8)	0	1 (3)
Inoue [30]	2019	Japan	NA	21 (70)	0	2 (6.6)
Furutachi [31]	2015	Japan	11	13 (68.4)	2 (10.5)	0
Shrestha [7]	2015	Germany	NA	NA	3 (6.8)	NA
Katayama [32]	2015	Japan	104	113 (100)	5 (4.4)	12 (10.6)
Xiao [33]	2014	China	27	NA	NA	1 (3.7)
Yang [34]	2014	China	28.5	68 (97.1)	NA	2 (2.4)
Shi [35]	2014	China	14	24 (100)	0	0
Ma [36]	2013	China	NA	NA	NA	NA
Roselli [10]	2013	USA	5	15 (88)	1 (6.0)	0
Hoffman [37]	2013	Germany	17	31 (100)	0	1 (3.1)
Uchida [38]	2011	Japan	74.3	76 (100)	6 (7.9)	5 (6.6)
Chen [39]	2010	China	30	23 (100)	1 (4.3)	2 (8.7)
Tsakagis [40]	2010	Germany	23	46 (94)	8 (13.5)	6 (9)
Pochettino [41]	2009	USA	30	23 (100)	1 (4.3)	2 (8.7)
Jakob [42]	2008	Germany	23	18 (90)	2 (10)	4 (20)

## Data Availability

Not applicable.
